# Use of a nested PCR-enzyme immunoassay with an internal control to detect *Chlamydophila psittaci *in turkeys

**DOI:** 10.1186/1471-2334-5-76

**Published:** 2005-09-26

**Authors:** Marnix Van Loock, Kristel Verminnen, Trudy O Messmer, Guido Volckaert, Bruno M Goddeeris, Daisy Vanrompay

**Affiliations:** 1 Department of Biosystems, Catholic University of Leuven, Kasteelpark Arenberg 30, 3001 Heverlee, Belgium; 2 Department of Molecular Biotechnology, Ghent University, Coupure Links 653, 9000 Gent, Belgium; 3 Department of Health and Human Services, National Center for Infectious Diseases, Centres for Disease Control and Prevention, Public Health Service, Atlanta, Georgia 30333, USA; 4 Department of Virology, Parasitology and Immunology, Ghent University, Salisburylaan 133, 9820 Merelbeke; Belgium

## Abstract

**Background:**

Laboratory diagnosis of *Chlamydophila psittaci*, an important turkey respiratory pathogen, is difficult. To facilitate the diagnosis, a nested PCR-enzyme immunoassay (PCR-EIA) was developed to detect the *Cp. psittaci *outer membrane protein A (*ompA*) gene in pharyngeal swabs.

**Methods:**

The fluorescein-biotin labelled PCR products were immobilized on streptavidin-coated microtiter plates and detected with anti-fluorescein peroxidase conjugate and a colorimetric substrate. An internal inhibition control was included to rule out the presence of inhibitors of DNA amplification. The diagnostic value of the *ompA *nested PCR-EIA in comparison to cell culture and a 16S-rRNA based nested PCR was assessed in pharyngeal turkey swabs from 10 different farms experiencing respiratory disease.

**Results:**

The sensitivity of the nested PCR-EIA was established at 0.1 infection forming units (IFU). Specificity was 100%. The *ompA *nested PCR-EIA was more sensitive than the 16S-rRNA based nested PCR and isolation, revealing 105 out of 200 (52.5%) positives against 13 and 74 for the latter two tests, respectively. Twenty-nine (23.8%) out of 122 *ompA *PCR-EIA negatives showed the presence of inhibitors of DNA amplification, although 27 of them became positive after diluting (1/10) the specimens in PCR buffer or after phenol-chloroform extraction and subsequent ethanol precipitation.

**Conclusion:**

The present study stresses the need for an internal control to confirm PCR true-negatives and demonstrates the high prevalence of chlamydiosis in Belgian turkeys and its potential zoonotic risk. The *ompA *nested PCR-EIA described here is a rapid, highly sensitive and specific diagnostic assay and will help to facilitate the diagnosis of *Cp. psittaci *infections in both poultry and man.

## Background

Avian chlamydiosis is caused by the obligate intracellular Gram-negative bacterium *Chlamydophila psittaci *(formerly *Chlamydia psittaci*). Currently, seven genotypes of *Cp. psittaci *are known to infect birds [1-3]. Avian chlamydiosis in birds is usually systemic and occasionally fatal. The clinical signs vary greatly in severity and depend on the species, age of the bird and the strain of *Cp. psittaci*. Avian chlamydiosis can produce lethargy, hyperthermia, abnormal excretions, nasal and eye discharges, and reduced egg production. Mortality rates range up to 30% [4]. Avian chlamydiosis occurs worldwide, with the incidence and distribution varying greatly with the species of bird and the serotype of the chlamydial organism. In the past, chlamydiosis in turkeys was thought to be limited to the United States and to free-ranging flocks. Most outbreaks in US turkeys were explosive, involving one or more flocks [5-10]. Nowadays, the increase in confinement-rearing of turkeys and the prevention of wild birds flying in and out the turkey houses seems to contribute to a decrease of severe outbreaks. Probably, the situation is comparable to the one in Europe where, at present, *Cp. psittaci *is nearly endemic in Belgian, German and probably French turkeys [11-13]. However, devastating, explosive outbreaks with high mortality rates occur occasionally, whereas present outbreaks are mostly characterized by respiratory signs without mortality [4]. Nevertheless, *Cp. psittaci *causes important economical losses as a primary pathogen and trough it's pathogenic interaction with other respiratory pathogens like the avian pneumovirus (APV) and *Ornithobacterium rhinotracheale *(ORT) [13]. *Cp. psittaci *is also a threat to public health as this zoonotic agent can infect humans and precautions should be taken when handling infected birds or contaminated materials [14-17]. Human infections are common following handling or processing of infected turkeys or ducks [2,7,8,18]. Most infections are through inhalation of infectious aerosols and subsequently processing plant employees, farm workers, veterinarians and poultry inspectors are at risk. However, personnel who were employed to further process turkey meat could also become infected [19].

Thus, diagnosis is essential. In contrast to cell culture and serology, antigen detection methods like micro-immunofluorescence and PCR provide a more rapid, specific and sensitive alternative for identification of *Cp. psittaci *infection. However, currently described PCR assays for birds use either labour intensive and/or insensitive post PCR detection methods. A PCR-enzyme immunoassay (PCR-EIA) would circumvent this problem. At the moment, we are not aware of a nested PCR- enzyme immunoassay (PCR-EIA) for demonstrating *Cp. psittaci *infection, although the method has been successfully used for *C. pneumoniae *detection in human respiratory specimens [20,21].

The objective of the present study was to develop and evaluate a rapid and simple EIA for semi-quantitative detection of the amplified *Cp. psittaci *outer membrane protein A (*ompA*) gene, included with an internal inhibition control to eliminate possible false positive results during field sample analysis.

## Methods

### Specimens

In the fall of 2001, 200 fattening turkeys from 10 different farms in Belgium (8 farms) or in Northern France (2 farms) were examined at slaughter for the presence of *Cp. psittaci*. All turkeys had been vaccinated against Newcastle disease (NCD) (Nobilis^® ^ND LaSota; Intervet International, Boxmeer, The Netherlands) and in 7 out of 10 farms turkeys had also been vaccinated against APV (Nobilis^® ^RTV; Intervet International). Farmers provided information about clinical symptoms throughout the rearing period. All farms had experienced one or more periods of respiratory disease.

Pharyngeal swabs were collected from 20 ad randomly selected turkeys on each turkey farm. Of each turkey there was taken 1 sample by using cotton tipped aluminium shafted swabs (Fiers, Kuurne, Belgium) in *Cp. psittaci *transport medium [22] consisting of: 0.2 M sucrose (VWR International, Haasrode, Belgium); 0.015 M Na_2_HPO_4 _(VWR International), 0.01 M NaH_2_PO_4 _(VWR International) and 20% inactivated foetal calf serum (Integro, Leuvenheim, The Netherlands). Swabs were shaken vigorously for 1 hour and centrifuged (10 min, 2790 × g, 4°C). One millilitre of supernatant was provided with 1% streptomycin sulphate (10 mg/ml; Invitrogen), 2% vancomycin (5 mg/ml; Glaxo Smith Kline) and 1.6% fungizone (250μl/ml; Invitrogen) and subsequently used for *Cp. psittaci *isolation.

### Generation of the internal control

**Figure 1:**
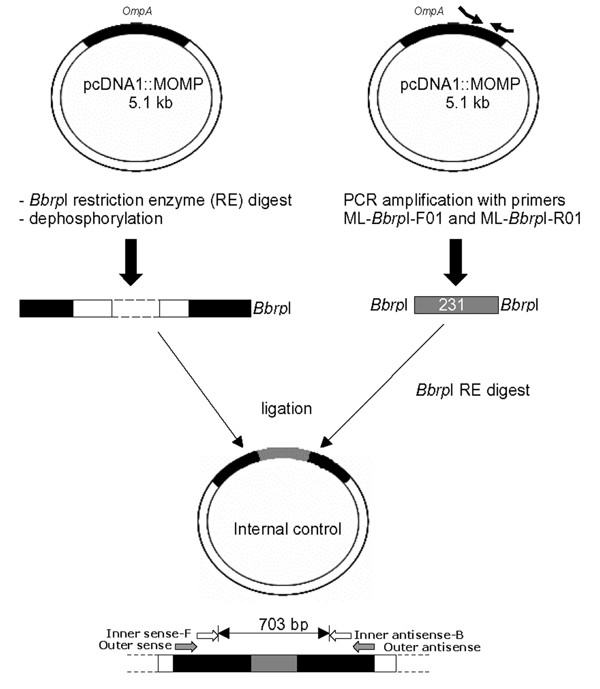
Generation of the internal control using primers ML-*Bbrp*1-F01 and ML-*Bbrp*1-R01.

**Figure 2:**
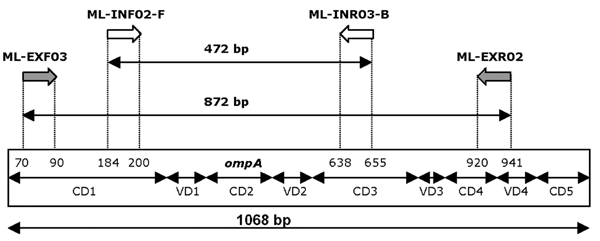
**Location of the outer and inner primers in the *ompA *gene.**Numbering according to *ompA *sequences in Genebank

The internal inhibition control was constructed starting from the pcDNA1 vector in which the *ompA *gene of a *Cp. psittaci *serovar D strain 92/1293 was inserted (Fig 1 &2) [23]. First, a fragment of 231 bp of the *ompA *gene was amplified using primers ML-BbrpI-F01 and ML-Bbrp1-R01 (table 1), which provided a BbrpI restriction site at their 5' end for subsequent cloning. The PCR reaction was performed using *Pfu *DNA polymerase in 50 μl reactions containing dNTP's (0.2 μM final concentration), *Pfu *buffer (10x), ML-BbrpI-F01 and ML-Bbrp1-R01 (0.5 μM final concentration), DMSO (7.5 %), *Pfu *DNA Polymerase (2.5 U, Stratagene, La Jolla, USA) and 2 μg plasmid DNA. After an initial denaturation at 95°C for 1 minute, 30 cycles of 30 seconds at 95°C, 30 seconds at 58°C and 1 minute at 72°C, followed by a final elongation at 72°C for 10 min, were performed. PCR products were subjected to electrophoresis on a 1.2% agarose gel stained with ethidium bromide and photographed under UV illumination. Size was determined using Smart Ladder (Eurogentec, Seraing, Belgium). PCR products were purified using the Qiaquick PCR Purification kit (Qiagen) and ligated into the pPCR-script™ Amp SK (+) vector (Stratagene, La Jolla, USA), as described by the manufacturer. Next, the vector was transformed into Epicurian coli XL10-Gold Kan ultracompetente cells (Stratagene) using heat shock. Clones were selected on Luria-Bertoni (LB) medium containing ampicilin (100 μg/ml) and grown in microtiter plates for 2 hours. The presence of the insert was confirmed by PCR clone analysis. Therefore, 5 μl of each clone was subjected to PCR in a 50 μl reaction mixture containing 50 mM KCl, 20 mM Tris-HCl (pH 8.3), 2 mM MgCl_2_, 0.1% Tween20, 200 μM each dNTP, 1.25 μM of each inner *ompA *primer (table 1) and 0.1 U SuperTaq polymerase (5 U/μl). After an initial denaturation at 95°C for 5 minutes, 20 cycles of one min at 95°C, two minutes at 55°C and three minutes at 72°C, with a final extension at 72°C for 5 min, were performed. To ensure PCR accuracy, the construct was sequenced using the ABI PRISM Bigdye™ Terminator Cycle Sequencing Ready Reaction Kit (ABI, Foster City, USA), following the manufacturers' manual. Sequencing samples were analyzed on the ABI PRISM 377 DNA sequencer (Perking Elmer).

**Table 1:** Oligonucleotides used in this study

**Oligonucleotide**	**Length (bp)**	**Sequence (5'-3')**
Sense outer 16 rRNA	18	ACG GAA TAA TGA CTT CGG
Anti-sense outer 16S rRNA	18	TAC CTG GTA CGC TCA ATT
Sense inner 16S rRNA	21	ATA ATG ACT TCG GTT GTT ATT
Anti-sense inner 16S rRNA	20	TGT TTT AGA TGC CTA AAC AT
ML-BbrpI-F01	29	GC*C ACG TG*C GTC TGC AAC ACT CAA ATA TC
ML-BbrpI-R01	28	GG*C ACG TG*C AGT TGT AAG AAG TCA GAG T
Sense outer o*mpA*	21	CCT GTA GGG AAC CCA GCT GAA
Anti-sense outer o*mpA*	22	GGT TGA GCA ATG CGG ATA GTA T
Fluorescein-sense inner o*mpA*	17	GCA GGA TAC TAC GGA GA
Biotin-antisense inner *ompA*	18	GGA ACT CAG CTC CTA AAG

Positive clones were grown overnight in 4 ml LB medium containing ampicilin (100 μg/ml) and subjected to the Qiaprep Spin Miniprep kit (Qiagen) to obtain purified plasmid. Using *Bbrp*I, the 231 bp fragment was cut out of the pPCR-script™ Amp SK (+) vector and ligated into the dephosphorylated *Bbrp*1 site, situated within the *ompA *gene of the pcDNA1::MOMP vector [23]. Thus, the *Bbrp*1 restriction site is located within the target sequence of the inner primers and subsequently, nested PCR amplification of the inhibition control resulted in a PCR product of 703 bp (fig. 1). *E. coli *strain MC1061/P3 was transfected by electroporation (Gene Pulser, Bio-Rad). Again, selected clones were subjected to PCR clone analysis to asses the presence of the insert and its sequence was determined by the dideoxy chain terminating method, as described above.

### Preparation of positive control DNA

*Cp. psittaci *strains genotype A to F strains were propagated in cycloheximide-treated BGM cells, as described elsewhere [24]. Bacteria were harvested at approximately 72 hours by disrupting the cells by subsequent freezing and thawing, followed by sonication and differential centrifugation (Urografin 76%). Purified elementary bodies were pelleted, washed, resuspended in sucrose-phosphate-glutamate buffer, and stored in aliquots at -70°C. For determination of bacterial titres, BGM monolayers grown on glass cover slips (Chlamydia Trac Bottles, International Medical) were infected and stained by the IMAGEN™ direct immunofluorescence assay [24]. Inclusion forming units (IFU) were determined by counting the numbers of inclusions cultured in duplicate in Chlamydia Trac Bottles using 10-fold serial dilutions of purified EBs. *Cp. psittaci *titres were expressed as IFU per millilitre and IFU quantitated in this manner were used as the positive-control DNA in PCR assays.

### *OmpA *nested PCR-EIA

Clinical specimens included pharyngeal swabs from turkeys taken at slaughter. These specimens as well as positive control DNA were prepared for ompA nested PCR-EIA by the STD DNA extraction method, performed as followed: Cp. psittaci suspensions or turkey specimens were pelleted at 13,000 x g, resuspended in 198 μl STD buffer (0.01 M Tris-HCl [pH 8.3], 0.05 M KCl, 0.0025 M MgCl2.6H20, 0.5% Tween20) and 2 μl proteinase K (20 mg/ml stock solution; Sigma Chemical Co.). The specimens were incubated at 56°C for one hour and subsequently heated at 100°C for 10 min. Samples for both PCR's were prepared in a class II laminar flow hood, and amplification and analysis of PCR products were performed in separate locations.

The nested *ompA *PCR-EIA was developed using external and internal primers (table 1) generating a biotin-fluorescein dual labelled internal PCR product of 472 bp. Both inner and outer sense primers are located within the first conserved domain (CD1) of *ompA*, whereas the inner anti-sense primer is located in CD3 and the outer anti-sense primers overlaps CD4 and variable domain 4 (fig. 2). First round PCR occurred in 50 mM KCl, 20 mM Tris-HCl (pH 8.3), 2 mM MgCl_2_, 0.1% Tween20, 200 μM each dNTP, 1.25 μM each external primer (table 1) and 0.1 U SuperTaq polymerase (5 U/μl). After an initial denaturation at 95°C for 5 minutes, 20 cycles of one min at 95°C, two min at 59°C and three min at 72°C, with a final extension at 72°C for 5 min were performed. Second round amplification was performed under similar conditions, using labelled internal primers (each 10 μM; table 1), adapted annealing temperature (47°C) and adapted number of cycles (25). Subsequently, nested PCR generated a fluorescein/biotin dual-labelled product of 472 bp. To minimize false-positive results, each step of the nested PCR was performed in physically separated places.

To allow colorimetric detection of the *ompA *PCR products, 50 μl of the PCR product diluted 1/10 in PBS supplemented with 3% BSA was transferred in duplicate to streptavidin-coated microtiter plates (2 μg/well for 3 hours at 37°C) and incubated at 37°C for one hour. Non-specific binding places were blocked overnight (4°C) with 5% BSA in PBS. Subsequently, the plates were washed twice with PBS and incubated (1 hour, 37°C) with a horseradish peroxidase labelled anti-fluorescein antibody (Invitrogen), diluted 1/1000 in PBS supplemented with 3% BSA. Following incubating and washing with PBS, the ABTS substrate solution (2,2' azino-di-3-ethylbenzothiazoline sulphonate, KPL) was added to the wells. Absorbencies were read at 450 nm after incubating for 30 minutes at 37°C (TiterTek MultiskanR Plus, MKII, TechGen Internatonal). Three positive controls consisting of serial 10-fold dilutions of PCR products generated from 5 IFU of *Cp. psittaci *and five negative controls (water) were included in each assay. Results were positive if the absorbance exceeded the cut off value of the mean of negative controls plus three times the standard deviation. Nested PCR-EIA negative samples were re-tested after adding 10 ng internal inhibition control and visualized by gel electrophoresis to assess possible inhibition.

The sensitivity of the PCR was evaluated by testing 10-fold serial dilutions of DNA extracted from purified elementary bodies of *Cp. psittaci *strains 92/1293 [2]. Next, diagnosis of six reference strains of *Cp. psittaci *serovars A-F was assessed (table 2). The specificity was determined by testing DNA extracted from other bacterial species commonly found in the avian respiratory tract and avian respiratory tract tissue, originating from *Cp. psittaci *negative specific pathogen free turkeys (CNEVA, Ploufragan, France). The following micro-organisms were tested for cross-reactivity in the PCR assay with both the first- and second-step PCR primers: *Acinetobacter *species, *Aspergillus flavus*, *Candida albicans*, *Enterococcus faecelis*, *Escherichia coli*, *Klebsiella species*, *Mycobacterium avium*, *Mycoplasma gallisepticum*, *Mycoplasma meleagridis*, *Ornithobacterium rhinotracheale, Pasteurella species*, *Proteus mirabilis*, *Pseudomonas species*, *Salmonella enteritidis*, *Salmonella gallinarum*, *Salmonella pullorum*, *Staphylococcus species, Streptococcus species *and *Xanthomonas maltophila*. A *Cp. psittaci *positive control of 1 IFU was included in every test to verify that the PCR was working.

**Table 2:** *Cp. psittaci *reference strains

**Strain**	**Year**	**Country**	**Host**	**Serovar**	**Reference**
VS 1	1985	USA, Georgia	*Amazona sp*.	A	[34]
CP3	1957	USA, Calofornia	*Columba livia*	B	[35]
GD	1960	Germany	*Anas platyrhynchos*	C	[36]
NJ1	1954	USA, New Jersey	*Meleagris gallopavo*	D	[37]
MN	1934	USA, California	*Homo sapiens*^a^	E	[38]
VS225	1991	USA, Texas	Parakeet	F	[39]

^a ^Probably originated from birds, isolated in ferrets from human [39].

### 16S-rRNA nested PCR – gel electrophoresis

The performance of the *ompA *nested PCR-EIA was compared to those of isolation and another nested PCR, targeting the 16S rRNA gene [25]. *Cp. psittaci *suspensions or turkey specimens were prepared for PCR by the QiaAmp Blood kit (Qiagen Inc., Chatworth, Califormia) adapted by [25]. Genus-specific first-step primers and species-specific second step primers generated PCR products of 436 bp and 127 bp, respectively (table 1). Amplification products were visualized by gel electrophoresis (1.5% Nusieve GTG agarose, FMC Bioproducts, Rockland, Maine). PCR negatives were spiked with 5 IFU of *Cp. psittaci *to control for the presence of inhibitors. The limit of detection of this 16S-rRNA-based PCR was 5 IFU as previously described [25]. Samples for both PCR's were prepared in a class II laminar flow hood, and amplification and analysis of PCR products were each performed in separate locations.

### Comparison to *Cp. psittaci *isolation

Pharyngeal swabs were examined for the presence of viable *Cp. psittaci *by isolation in cycloheximide-treated Buffalo Green Monkey (BGM) cells. Swabs were shaken at 4°C for 1 hour and centrifuged (10 minutes, 2790 × g, 4°C). The supernatant was used for *Cp. psittaci *isolation in BGM cells and subsequent identification using the IMAGEN™ direct immunofluorescence assay (DakoCytomation, Denmark), as previously described [24]. All inoculated monolayers were stained at 6 days post inoculation. Inclusion-negative cultures were passaged once. After adding an equal volume of sucrose phosphate glutamate (SPG; [25]) and freezing at -80°C, cultures were thawed, cell suspensions were sonicated and centrifuged once (2000 x g). Supernatant was inoculated in duplicate onto new BGM monolayers as described elsewhere [24]. Staining was performed at 3, and if negative at 6 days post inoculation.

### Validation

A specimen was considered positive if culture positive. In addition, a culture-negative, but 16S rRNA-based PCR positive specimen was considered to be a true positive only if it could be verified by *ompA*-based PCR.

## Results

### Development of the nested PCR-EIA

**Figure 3:**
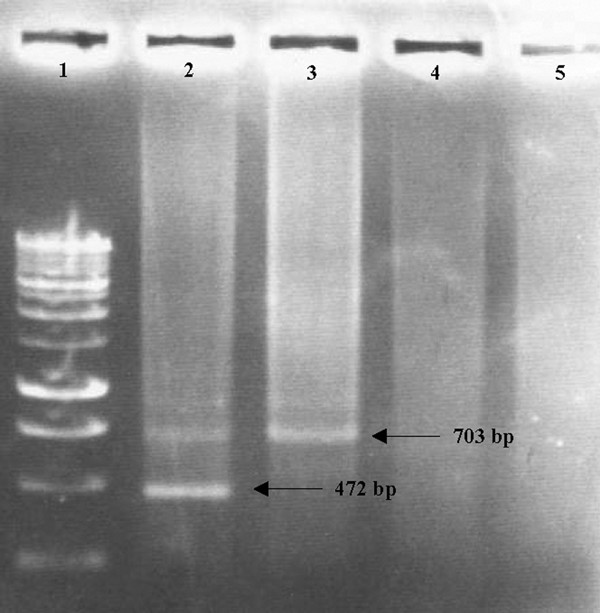
**Different conditions observed in the nested PCR-EIA analysis of *Cp. psittaci *in turkey field samples. **Lane 1: molecular marker (BenchTop 1 kb DNA ladder; Promega); lane 2: *Cp. psittaci *positive sample, showing one band (472 bp) diagnostic for *Cp. psittaci *and a band (703 bp) for the internal inhibition control; lane 3: *Cp. psittaci *negative sample, showing only the internal inhibition control band; lane 4: a sample with inhibitory substances lacking both the *Cp. psittaci*-specific band and the internal inhibition control band; lane 5: negative control, free from *Cp. psittaci *DNA and internal control DNA.

Optimizing PCR conditions was performed using STD extracted DNA of *Cp. psittaci *serovar D strain 92/1293. Initial PCR with temperature gradients were performed with either inner or outer primer sets separately to determine optimal annealing temperature for both primer sets. The optimal annealing temperature for outer and inner primer sets was determined at 59°C and 47°C, respectively (data not shown). Next, optimal primer dilutions were tested to obtain a single band as nested PCR product, after visualization on 1.2% agarose gel (fig. 3). Hereto, external primers were used at 0.625 μM and internal primers at 10 μM. First and second round PCR amplification with the outer and inner primers resulted in PCR products of 872 bp and 472 bp, respectively. After amplification biotinylated PCR products were immobilized to streptavidin-coated microtiter wells and detected with anti-fluorescein peroxidase conjugate and a colorimetric substrate. Next, optimal enzyme immunoassay conditions were realised, among others by diluting the dual labelled PCR product 1/10 in dilution buffer (PBS + 3% BSA + 2% IgG free horse serum). All incubation steps and reaction components of this EIA were optimized prior to use with pharyngeal swabs.

### Sensitivity

**Figure 4:**
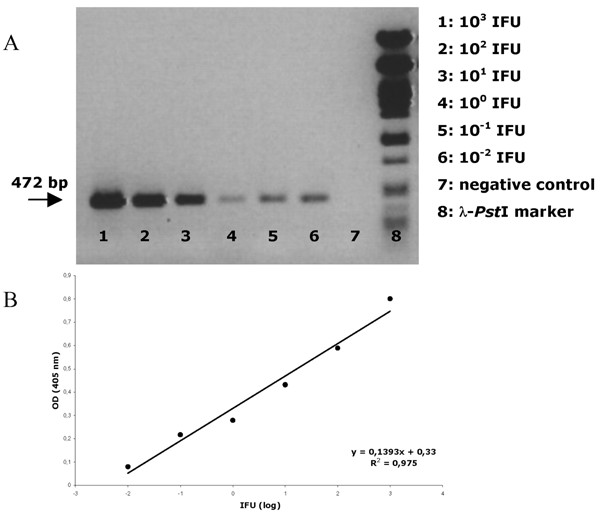
**(A) Nested PCR-EIA analysis on a tenfold serial dilution of *Cp. psittaci *strain 92/1293. (B) Visualisation of the tenfold serial dilution on a 1.2% agarose gel following nested PCR. **10^3 ^IFU (lane 1) until 10^-2 ^IFU (lane 6). Lane 7 shows the PCR results on a negative control. Lane 8: Phage lambda *Pst*I fragments as size marker.

Following definition of optimal reagent and reaction conditions, sensitivity and specificity of the *ompA *nested PCR was determined. The STD DNA-extraction was performed on 10^8 ^IFU and tenfold dilutions of the purified DNA were subjected to the nested PCR and visualised on a 1.2% agarose gel. This resulted in a final nested PCR product of 472 bp and a detection limit of 10^-2 ^IFU (fig. 4A). Subjecting the dual-labelled nested PCR product to the EIA, resulted also in a detection limit of 10^-2 ^IFU (fig. 4B) and there was a linear relationship between the measured absorbance and the tenfold dilution series. However, when the tenfold dilution series of *Cp. psittaci *elementary bodies was made prior to the STD DNA-extraction, sensitivity decreased one log to 10^-1 ^IFU. Similarly, when field samples, which tested negative by nested PCR, were spiked with the serial tenfold dilutions, sensitivity decreased to 10^-1 ^IFU.

Amplification of chlamydial DNA and the internal inhibition control was achieved with the *ompA *inner and outer primer sets, as the additional DNA fragment for the inhibition control was inserted within the target sequence of the *ompA *inner primer set. Therefore, the nested PCR amplification of *Cp. psittaci *cultures or inhibitory substance-free clinical specimens, which were positive for *Cp. psittaci *exhibited two bands on ethidium-stained agarose gel electrophoresis: one band (472 bp) diagnostic for *Cp. psittaci *and a control band (703 bp) for the internal inhibition control (fig 3). Inhibitory substance-free clinical specimens negative for *Cp. psittaci *contained only the control band, indicating that no detectable inhibitors were present and that biochemical conditions were optimal for PCR amplifications. When inhibitory substances were present in field samples, no bands were detected on ethidium-stained agarose gel. Adding 10 ng of inhibition control to the nested PCR mixture was determined as the optimal condition to assess inhibition in field samples (fig 3).

### Specificity

The nested PCR-EIA was able to detect al 6 tested *Cp. psittaci *reference strains (table 2), whereas strains of *Chlamydia trachomatis*, *Chlamydophila pneumoniae, Chlamydophila abortus and Chlamydophila felis *remained undetected. Furthermore, avian respiratory tract tissue originating from *Cp. psittaci *negative specific-pathogen-free turkeys (CNEVA, Ploufragan, France) and a wide range of non-chlamydial bacteria were tested and showed no cross-reactivity with: *Acinetobacter *species, *Aspergillus flavus*, *Candida albicans*, *Enterococcus faecelis*, *Escherichia coli*, *Klebsiella species*, *Mycobacterium avium*, *Mycoplasma gallisepticum*, *Mycoplasma meleagridis*, *Ornithobacterium rhinotracheale, Pasteurella species*, *Proteus mirabilis*, *Pseudomonas species*, *Salmonella enteritidis*, *Salmonella gallinarum*, *Salmonella pullorum*, *Staphylococcus species, Streptococcus species *and *Xanthomonas maltophila*. A *Cp. psittaci *positive control of 1 IFU was included in every test to verify that the PCR was working.

### Analysis of pharyngeal specimens

Two hundred turkeys from 10 different farms in Belgium (8 farms) or in Northern France (2 farms) were examined at slaughter for the presence of *Cp. psittaci*. All farms had experienced one or more periods of respiratory disease. All samples have been analysed for the presence of *Cp. psittaci *by isolation in BGM cells, nested PCR-EIA and 16S rRNA nested PCR (table 3). The pharyngeal swabs were inoculated onto cycloheximide-treated BGM cells. *Cp. psittaci *was isolated from 54 specimens (27%) after the first inoculation and from 20 additional samples (10%) following one passage. Thus, 74 out of 200 (37%) specimens revealed to be culture positive. They were all confirmed as culture positive by *ompA *nested PCR-EIA analysis of the infected BGM monolayers. One hundred and twenty six samples remained negative, notwithstanding an additional 6 days passage on BGM cells. *OmpA *nested PCR-EIA was able to detect chlamydial DNA successfully in 105 on 200 (52.5%) pharyngeal swabs. However, 29 out of 122 (23.8%) PCR-EIA negatives clearly demonstrated inhibition showing no internal control band on the agarose gel. Seventeen out of 29 samples containing inhibitors became positive after prior 1/10 dilution in PCR buffer. Specimens that still showed inhibition were subjected to phenol-chloroform extraction and ethanol precipitation to further purify the DNA and were retested. Ten tested positive for *Cp. psittaci*, but 2 of 29 specimens continued to show inhibition. Thus, finally 105 out of 200 (52.5%) pharyngeal swabs tested positive by the *ompA *nested PCR-EIA. Surprisingly, the 16S rRNA-based PCR could only confirm 13 out of 105 *ompA *PCR positives revealing a total of 6.5% positive turkeys. Nine of these 13 positives could be confirmed by isolation while the remaining 4 were negative by isolation but positive by *ompA *PCR, indicating that they were true positives.

**Table 3:** Results of nested PCR's on turkey pharyngeal specimens compared to isolation as a reference test

**Isolation**	**N**	***OmpA PCR***		**16S rRNA PCR**	
		**positive**	**negative**	**positive**	**negative**
Negatives	126	31	95	4	122
Positives	74	74	0	9	65
Total	200	105	95	13	187

Referring to isolation as a reference, all 10 examined farms were *Cp. psittaci *positive at slaughter. The same was true when looking at the *ompA *nested PCR results. Although apparently less sensitive, the 16S rRNA PCR detected *Cp. psittaci *in 50% of the examined farms.

## Discussion

A nested PCR-EIA based on the detection of the *ompA *gene was developed and evaluated for the diagnosis of chlamydiosis in turkeys. Nested PCR resulted in a 5' fluorescein and 3' biotin labelled *ompA *fragment of 472 bp which was subsequently detected in an enzyme immunoassay. Although the designed inner and outer anti-sense primers showed few nucleotide mismatches as compared to the *ompA *sequences of *Cp. psittaci *genotype C, D and F reference strains, amplification of *Cp. psittaci *genotypes A to F strains consistently resulted in the anticipated nested PCR-EIA product. The nested PCR-EIA was 100% specific as all *Cp. psittaci *genotypes were detected, but no *C. trachomatis, C. pneumoniae, C. abortus *or *C. felis *DNA. Additionally, no cross-reactivity was observed with other bacterial respiratory pathogens commonly found in the avian respiratory tract or with turkey respiratory tract DNA.

Nested PCR was chosen in order to obtain high sensitivity and specificity. Amplification of internal control DNA helped us in confirming true-negative PCR results by ruling out the presence of inhibitors of DNA amplification. Adding 10 ng of the internal inhibition control did not comprise sensitivity, as 10^-2 ^IFU of all *Cp. psittaci *genotype reference strains was detected. However, when field samples, which tested negative in the nested PCR-EIA were spiked with the tenfold DNA dilutions, sensitivity decreased to 10^-1 ^IFU, probably due to the higher amount of background DNA.

In the present study, 200 commercial turkeys, originating from 10 different farms in Belgium (8 farms) or Northern France (2 farms) were sampled at slaughter to examine for the presence of *Cp. psittaci*. Isolation in BGM cells revealed 74 (37%) positives. Application of the *ompA *nested PCR-EIA on pharyngeal DNA could confirm all culture positive results. Moreover, the *ompA *nested PCR-EIA detected 31 additional positives, resulting in a total of 105 (52.5%) *Cp. psittaci *positive turkeys. However, 29 (23.8%) of the 105 PCR-EIA positives were initially negative by the EIA and during retesting, when the internal control was added to the PCR mix, they demonstrated inhibition lacking the internal control band on an EtBr stained agarose gel. Yet, 17 samples became positive after prior 1/10 dilution of the specimen in PCR buffer. Moreover, after phenol-chloroform extraction and ethanol precipitation all but two of the sample became positive. Those two samples could not be diagnosed by the *ompA *nested PCR-EIA, as inhibitory substances could not be removed. Culture and 16S rRNA nested PCR for those 2 samples were also negative. However as the latter two tests have shown to be less sensitive and consequently, the presence or absence of *Cp. psittaci *in these 2 samples cannot be conclude. The present results clearly demonstrate the importance of using an internal control to help identify true-negatives when examining turkey pharyngeal swabs, as inhibition of DNA amplification seem to occur rather frequently in these specimens. Notwithstanding the presence of polymerase inhibitors, pharyngeal swabs still remain the first choice for sampling live birds. Pharyngeal specimens are preferred as cloacal shedding of *Cp. psittaci *is intermittent and, in contrast, the respiratory tract appears to be the last system to be cleared of infection. Furthermore, pathogenesis of *Cp. psittaci *revealed that lateral nasal glands can be infected for a extended period [26]. Secretions of these glands function to keep the mucosa moist and drainage of infected secretions into the pharyngeal cavity can serve as source for *Cp. psittaci*. Also secretions from the lung are expelled into the pharyngeal area [27].

Surprisingly, the 16S rRNA-based PCR could only confirm 13 out of 105 *ompA *nested PCR-EIA positives revealing only 6.5% positive turkeys. Discrepant results were probably not due to different extraction methods, as 30 16S-rRNA negative samples remained negative even after using the STD extraction method as for the *ompA *- based nested PCR.

The PCR-EIA turned out to be more sensitive than isolation in cell culture and more sensitive than the 16S rRNA-based nested PCR. The 16S-rRNA PCR primers have already been shown to be sensitive (5 IFU) and specific [25]. The *ompA *nested PCR-EIA is approximately 50 times more sensitive than the 16S-rRNA based PCR. The sensitivity of the nested PCR-EIA was also superior to isolation of *Cp. psittaci *in cell culture, which is in agreement with other reports [28-30]. Moreover, the nested PCR-EIA is easy, rapid, and less labour-intensive than isolation and non-viable *Chlamydiaceae *can be detected, due to the relative high stability of DNA. This allows less stringent demands on collection, transportation and storage of the samples, making the nested PCR-EIA an ideal diagnostic method for monitoring turkey flocks during processing.

Referring to isolation as a reference, all 10 examined farms were *Cp. psittaci *positive at slaughter. The same was true when looking at the *ompA *nested PCR results. Although apparently less sensitive, the 16S rRNA PCR still detected *Cp. psittaci *in 50% of the examined farms. Results are in concordance with previous reports, demonstrating the high prevalence of *Cp. psittaci *in Belgian turkeys [13]. Public health is here of concern, as poultry workers, veterinary surgeons and slaughterhouse employees are at risk of becoming infected by this zoonotic agent [9,15,16,19,31-33]. In the present study, 37% of the turkeys were still shedding infectious *Cp. psittaci*, when transported to the slaughterhouse posing a threat to human health. Thus, the presented *ompA *nested PCR-EIA will help to facilitate the diagnosis of *Cp. psittaci *infections in both poultry and man.

## Competing interests

The author(s)declare that they have no competing interests

## Authors' contributions

Marnix Van Loock and Kristel Verminnen have made substantial contributions to conception, design, acquisition of data, analysis and interpretation of data. Trudy Messmer made substantial contributions to acquisition of data, analysis and interpretation. Guido Volckaert, Bruno Goddeeris and Daisy Vanrompay have been involved in revising the manuscript critically for important intellectual content.

## Pre-publication history

The pre-publication history for this paper can be accessed here:


